# Object-based multiscale segmentation incorporating texture and edge features of high-resolution remote sensing images

**DOI:** 10.7717/peerj-cs.1290

**Published:** 2023-03-15

**Authors:** Xiaole Shen, Yiquan Guo, Jinzhou Cao

**Affiliations:** College of Big Data and Internet, Shenzhen Technology University, Shenzhen, China

**Keywords:** Texture feature, Edge intensity, Time-frequency analysis, Multiscale segmentation, Object-based

## Abstract

Multiscale segmentation (MSS) is crucial in object-based image analysis methods (OBIA). How to describe the underlying features of remote sensing images and combine multiple features for object-based multiscale image segmentation is a hotspot in the field of OBIA. Traditional object-based segmentation methods mostly use spectral and shape features of remote sensing images and pay less attention to texture and edge features. We analyze traditional image segmentation methods and object-based MSS methods. Then, on the basis of comparing image texture feature description methods, a method for remote sensing image texture feature description based on time-frequency analysis is proposed. In addition, a method for measuring the texture heterogeneity of image objects is constructed on this basis. Using bottom-up region merging as an MSS strategy, an object-based MSS algorithm for remote sensing images combined with texture feature is proposed. Finally, based on the edge feature of remote sensing images, a description method of remote sensing image edge intensity and an edge fusion cost criterion are proposed. Combined with the heterogeneity criterion, an object-based MSS algorithm combining spectral, shape, texture, and edge features is proposed. Experiment results show that the comprehensive features object-based MSS algorithm proposed in this article can obtain more complete segmentation objects when segmenting ground objects with rich texture information and slender shapes and is not prone to over-segmentation. Compare with the traditional object-based segmentation algorithm, the average accuracy of the algorithm is increased by 4.54%, and the region ratio is close to 1, which will be more conducive to the subsequent processing and analysis of remote sensing images. In addition, the object-based MSS algorithm proposed in this article can effectively obtain more complete ground objects and can be widely used in scenes such as building extraction.

## Introduction

Multiscale segmentation (MSS) is vital for high-resolution remote sensing image analysis (Zhang et al., 2014; Johnson & Xie, 2013; Zhang, Xiao & Feng, 2017). The MSS algorithm can establish a strong foundation for further image analysis, such as terrain classification, target recognition, information extraction, *etc.* (Johnson et al., 2015; Zhang et al., 2015; Ma et al., 2018; Johnson, Tateishi & Hoan, 2013). The MSS is executed based on a variety of low-level features of the image, such as spectral feature and shape feature. The features of the image objects are comprehensively utilized and analyzed, and then the image objects are identified and processed to achieve further applications such as object detection. The MSS algorithm using spectral features and shape features has been widely studied. With the development of the MSS algorithm, scholars continue to integrate new features into the algorithm.

In recent years, some scholars have focused on improving and optimizing MSS algorithms by incorporating spectral and texture features. Wang & Li (2014) proposed a segmentation method of high spatial resolution remote sensing images based on the fusion of spectral, texture, and shape features. They used nonsubsampled contourlet transform to obtain texture features of the regions, and proposed an integrated region merging criterion by combining the texture, spectral, and shape features. Di et al. (2017) proposed a method for the multi-scale segmentation of high-resolution remote sensing images by integrating multiple features. They used Canny operator to extract edge information, and proposed the adaptive rule of Mumford-Shah region merging combination with spectral and texture information for segmentation. Fu et al. (2018) proposed a fast and efficient framework for multiscale and multifeatured hierarchical image segmentation. The algorithm successfully integrate spectral information, texture information, and structural information from a small number of superpixels to enhance expressiveness.  Lu, Wang & Yin (2019) proposed a super-pixel method based on the simple linear iterative clustering for remote sensing images segmentation at high spatial resolution. They modified the algorithm by incorporating grey-level co-occurrence matrix texture with color features and improved the measure approach with weighted distance of texture and color similarity.  Hu et al. (2021) proposed an MSS algorithm using a scale-sets structure, in which each segment is represented as a node of the hierarchy. In the algorithm, segments are described using spectral, textural, and geometric features, and then are classified using a random forest classifier for further applications.

Additionally, some algorithms and frameworks that adopt edge features have also been further developed. Ji, Zhang & Zhang (2013) proposed an improved edge linking method for segmentation of remotely sensed imagery. This method generated edges using the heuristic search method, which links all the initial edge points based on the gradient strength, gradient direction, and edge direction. Precise, continuous, and one-pixel-wide edges were produced for edge-based segmentation. Zhang et al. (2013) proposed a boundary-constrained multi-scale segmentation method. In this method, to improve the accuracy of object boundaries, the property of edge strength is used as a merging criterion. Wang & Li (2014) proposed a hard-boundary constraint and two-stage merging method for remote sensing image segmentation. Edge-constrained watershed segmentation and edge allocation were used to obtain initial small segments. In the first stage, they proposed a hard-boundary ratio to control the merge effectively. The second non-constrained merging stage is conducted on the initial object primitives, which results in final segmentation. Zhang, Xiao & Feng (2014) proposed a fast hierarchical segmentation method for high-resolution remote sensing images. They proposed an adaptive edge penalty function to formulate the merging criterion, serving as a semantic factor, which can help to remove meaningless weak edges within objects. Su (2019) proposed a new segmentation technique by fusing a region-merging method with an unsupervised segmentation evaluation technique called under- and over-segmentation aware, which is improved using edge information. The research on edge features has further improved the accuracy of the traditional MSS algorithm.

Scholars have also adopted methods such as optimization theory, semantics, and deep learning to broaden the research scope of MSS algorithms. Zhang, Tong & Chen (2012) made use of self-organizing, adaptive, and self-learning characteristics of evolutionary computation to automatically optimize the parameters of the multi-scale segmentation algorithm according to the evaluation of segmentation results. Shen et al. (2019) proposed an adaptive parameter optimization method for MSS. To find the optimal scale of objects, a local spectral heterogeneity measure was applied by calculating the spectral angle between inter and intra objects. Jiang, Huo & Feng (2020) proposed an optimal segmentation algorithm. This algorithm combines the principal component analysis (PCA) method with fuzzy c-means (FCM) method. Zhao et al. (2022) proposed an end-to-end attention-based semantic segmentation network (SSAtNet). In the encoder phase, they designed a more effective ResNet-101 backbone to capture detailed features. The aforementioned research further laid the foundation for the development of the MSS algorithm.

Much of the extant research has mainly focused on spectral features, shape features, texture features and edge features. However, less research has considered all these features. To address this problem, we start from the aspect of texture feature and propose the concept of texture heterogeneity. Then, combining texture feature with spectral feature and shape feature, a set of control experiments is designed. The results show that the algorithm greatly improves the accuracy of the MSS algorithm. Next, the concept of edge intensity is proposed and combined with texture, spectrum, and shape feature. Two sets of control experiments are designed, and the results show that the algorithm further improves the segmentation accuracy of the multiscale and multifeatured algorithm. The rest of the article is organized as follows: In the METHODS section, we review traditional MSS methods and object-based segmentation methods and describe the remote sensing image texture feature. Then, we propose the concepts of texture heterogeneity, edge intensity, and region ratio and apply them to traditional MSS methods in the RESULTS. At the same time, we created three groups of control experiments to test the accuracy of the proposed algorithm and obtain the comparison results with the traditional algorithm. Finally, the full text is summarized in the CONCLUSION.

## Methods

### Multiscale image segmentation with color and shape features

MSS is used in the famous remote sensing image analysis software eCognition Developer, which is produced by Definiens (Munich, Germany) and serves as the core algorithm for object-based image analysis. The basic idea of MSS is that images in nature conform to the fractal theory: that is, a typical structure will appear on different scales of an image, and show a certain degree of irregularity and self-similarity on various scales. The fractal feature is particularly obvious in remote sensing images. Mountains and rivers in the images all have fractal features. For example, in remote sensing images of urban areas, dense building areas will present: at a fine scale (small scale), buildings and building shadows form light and dark texture features; at a medium scale, buildings and the open space between the buildings form a texture feature of light and dark; and at a coarse scale (large scale), the regularly arranged buildings and buildings also form a periodic texture feature. This self-similarity feature is manifested in the fractal feature of remote sensing images.

**Figure 1:**
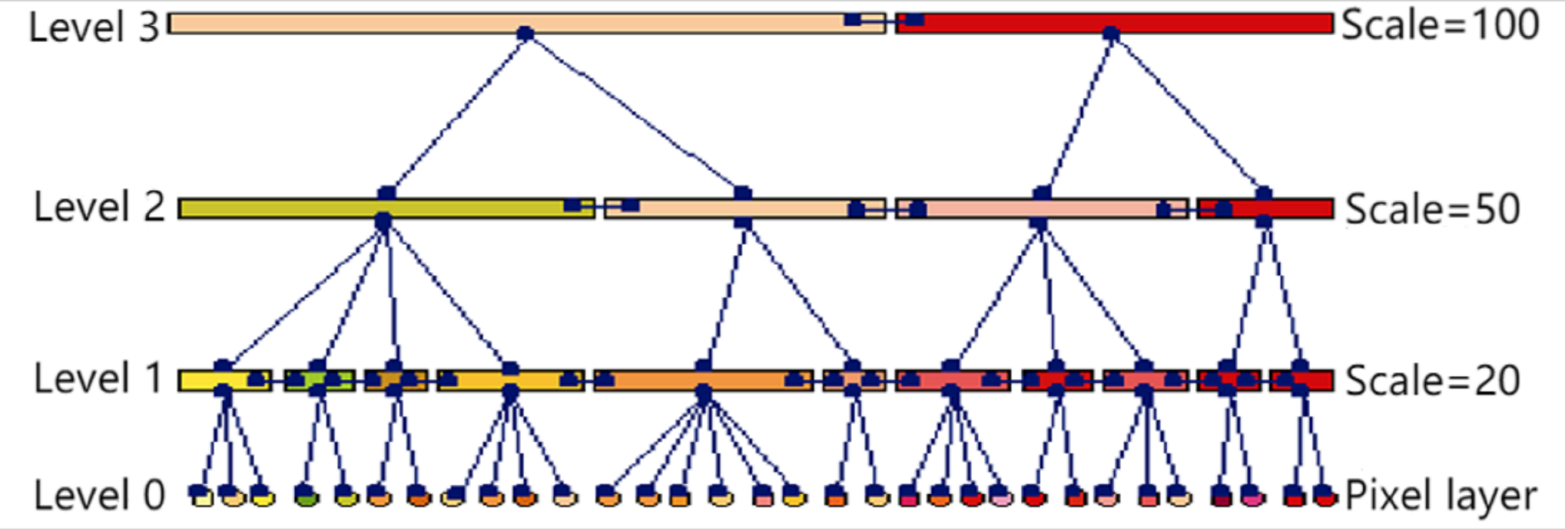
Image object hierarchical network diagram.

In many different segmentation scales, the MSS yields the segmentation result of the image at the corresponding scale, *i.e.,* the image object collection. Connecting image objects of different scales in the same area in the image forms a hierarchical network of image objects, as shown in Fig. 1. In the hierarchical network, each image object not only saves its attribute information, such as spectral mean, variance, area, perimeter, edge, and other information, but also saves its adjacent object information and affiliation (child object and parents object). When comparing two image objects with similar attributes in a remote sensing image, it is particularly important to analyze their scale level and the semantic information between adjacent objects and subordinate objects.

The MSS is a bottom-up region merging algorithm. The algorithm adopts the merging criterion of the least increase in heterogeneity as the regional merging strategy. At the beginning of the segmentation, the algorithm regards each pixel in the image as a minimum image object and finds the local best mutual matching object for merging through the corresponding merging criteria. The algorithm regards the heterogeneity growth threshold as the scale parameter in image segmentation and, by setting different scale parameters, the image can be segmented at multiple scales.

In the MSS, the degree of object heterogeneity represents the degree of heterogeneity of the internal attributes of an image object. The algorithm uses the growth value of the object’s heterogeneity and its threshold as the criteria for judging whether the object is merged and with whom. The measurement method of image object heterogeneity is the core of the whole algorithm. The algorithm considers the spectral feature and shape feature of the image and proposes two standards for measuring the heterogeneity of image objects: spectral heterogeneity and shape heterogeneity. Spectral heterogeneity describes the degree of heterogeneity of the spectrum inside an image object. The definition of spectral heterogeneity *h*_*color*_ is as follows:

(1) \begin{eqnarray*}~{h}_{\text{color}}=\sum _{c}{\omega }_{c}\cdot {\sigma }_{c}.\end{eqnarray*}

*c* is spectral channel and *ω*_c_ represents weighting factor of *c*-th band, which is supposed to meet 0 ≤ *ω*_*c*_ ≤ 1 and ∑_*c*_*ω*_*c*_ = 1.*σ*_*c*_ represents the standard deviation of spectral values in the *c*-th band. For the shape feature of the remote sensing image object, the algorithm proposes two criteria for shape heterogeneity: compact heterogeneity and smooth heterogeneity. Compact heterogeneity describes the compactness in the shape of image objects. The closer the shape of the object is to a circle, the greater the compactness and the smaller the value of the compact heterogeneity. *h*_cmpct_ can be defined as follows:

(2) \begin{eqnarray*}{h}_{\text{cmpct}}= \frac{l}{\sqrt{n}} .\end{eqnarray*}

*n* is the area of the image object (number of pixels), and *l* is the perimeter of the image object. Smoothing heterogeneity describes how smooth the image object is in shape. The closer the shape of the object is to a rectangle, the smoother the edge of the object, and the smaller the value of the smoothing heterogeneity. *h*_smooth_ can be defined as follow:

(3) \begin{eqnarray*}{h}_{\text{smooth}}= \frac{l}{b} .\end{eqnarray*}

In the formula, *l* represents the perimeter of the image object, and *b* represents the perimeter of the smallest circumscribed rectangle of the image object. In raster images, the algorithm believes that the smallest bounding rectangle can be replaced, approximately, by the bounding box of the object. Therefore, when the calculated image object *Obj1* and the image object *Obj2* are merged into the image object *Obj*_*Merge*_, the algorithm defines the growth value *f* of the overall object heterogeneity as the weighted sum of the spectral heterogeneity growth value Δ*h*_color_ and the shape heterogeneity growth value Δ*h*_shape_:

(4) \begin{eqnarray*}f={\omega }_{\text{color}}\cdot \Delta {h}_{\text{color}}+{\omega }_{\text{shape}}\cdot \Delta {h}_{\text{shape}}.\end{eqnarray*}

*ω*_*color*_ and *ω*_*shape*_ are the spectral weighting factor and shape weighting factor, respectively, which must meet 0 ≤ *ω*_*color*_ ≤ 1, 0 ≤ *ω*_*shape*_ ≤ 1 and *ω*_color_ + *ω*_shape_ = 1. The growth value Δ*h*_color_ of spectral heterogeneity is defined as:

(5) \begin{eqnarray*}\Delta {h}_{\text{color}}=\sum _{c}{\omega }_{c}({n}_{\text{Merge}}\cdot {\sigma }_{c}^{\text{Merge}}- \left( {n}_{\text{Obj1}}\cdot {\sigma }_{c}^{\text{Obj1}}+{n}_{\text{Obj2}}\cdot {\sigma }_{c}^{\text{Obj2}} \right) ).\end{eqnarray*}

In the formula, *c* represents the band, and *ω*_*c*_ represents the weighting factor of the c-th spectral band, which must meet 0 ≤ *ω*_*c*_ ≤ 1 and ∑_*c*_*ω*_*c*_ = 1.*n*_Obj1_, *n*_Obj2_, *n*_Merge_ is the area (that is, the number of pixels) of the object *Obj1*, the object *Obj2*, and the combined object. ${\sigma }_{c}^{\text{Obj1}}$, ${\sigma }_{c}^{\text{Obj2}}$, ${\sigma }_{c}^{\text{Merge}}$ is the standard deviation of the spectral value of the object *Obj1*, the object *Obj2*, and the combined object in the *c*-th band. The algorithm defines the shape heterogeneity growth value Δ*h*_shape_ as the weighted sum of the compact heterogeneity growth value Δ*h*_cmpct_ and the smooth heterogeneity growth value Δ*h*_smooth_:

(6) \begin{eqnarray*}\Delta {h}_{\text{shape}}={\omega }_{\text{cmpct}}\cdot \Delta {h}_{\text{cmpct}}+{\omega }_{\text{smooth}}\cdot \Delta {h}_{\text{smooth}}.\end{eqnarray*}

*ω*_*cmpct*_ and *ω*_*smooth*_ represent the compact weight factor and smooth weight factor, respectively, which must meet 0 ≤ *ω*_*cmpct*_ ≤ 1, 0 ≤ *ω*_*smooth*_ ≤ 1, and *ω*_cmpct_ + *ω*_smooth_ = 1. Compact heterogeneity growth value Δ*h*_cmpct_ and smooth heterogeneity growth Δ*h*_smooth_ are defined as:

(7) \begin{eqnarray*}\Delta {h}_{\text{cmpct}}={n}_{\text{Merge}}\cdot \frac{{l}_{\text{Merge}}}{\sqrt{{n}_{\text{Merge}}}} - \left( {n}_{Obj1}\cdot \frac{{l}_{Obj1}}{\sqrt{{n}_{Obj1}}} +{n}_{Obj2}\cdot \frac{{l}_{Obj2}}{\sqrt{{n}_{Obj2}}} \right) \end{eqnarray*}

(8) \begin{eqnarray*}\Delta {h}_{\text{smooth}}={n}_{\text{Merge}}\cdot \frac{{l}_{\text{Merge}}}{{b}_{\text{Merge}}} - \left( {n}_{Obj1}\cdot \frac{{l}_{Obj1}}{{b}_{Obj1}} +{n}_{Obj2}\cdot \frac{{l}_{Obj2}}{{b}_{Obj2}} \right) .\end{eqnarray*}

In the above two formulas, *n*_Obj1_, *n*_Obj2_, *n*_Merge_ is the area (the number of pixels) of the object *Obj1*, the object *Obj2*, and the combined object, respectively. *l*_Obj1_, *l*_Obj2_, *l*_Merge_ is the perimeter of the object *Obj1*, the object *Obj2*, and the merged object, respectively. *b*_Obj1_, *b*_Obj2_, *b*_Merge_ is the perimeter of the smallest bounding rectangle of the object *Obj1*, the object *Obj2*, and the merged object, respectively. It can be seen from the above description that the image segmentation result obtained by the MSS meets the minimum sum of the weighted heterogeneity of the image object on a certain scale *s*. At this scale, the image objects in the segmentation result are small enough in attribute differences, and the attribute differences between image objects are large enough.

### Multiscale segmentation incorporating texture feature

The object-based image analysis method takes the image object as the basic unit. The segmentation results of images at different scales are obtained through the bottom-up region merging process. The segmentation process considers the spectral and shape features of the image object. The texture homogeneity between adjacent pixels in the image is not considered. Based on the analysis image texture, we propose a remote sensing image texture feature descriptor based on time-frequency analysis. Furthermore, this section constructs the texture heterogeneity measure of the image object based on this method, and an MSS algorithm for object-based remote sensing images combined with texture feature is proposed.

#### Texture feature descriptor

When humans observe an image, they will find that although some ground objects do not have regularity in a local area, they will show a certain law when viewed as a whole. This feature of local irregularity and overall regularity is called texture. Humans have a clear perceptual understanding of the texture feature in images, but it is difficult to determine a unified mathematical definition. Currently, the commonly used methods of texture feature description can be divided into four categories: statistics-based methods, structure-based methods, model-based methods, and time-frequency analysis-based methods (Zhang & Tan, 2002).

**Figure 2:**
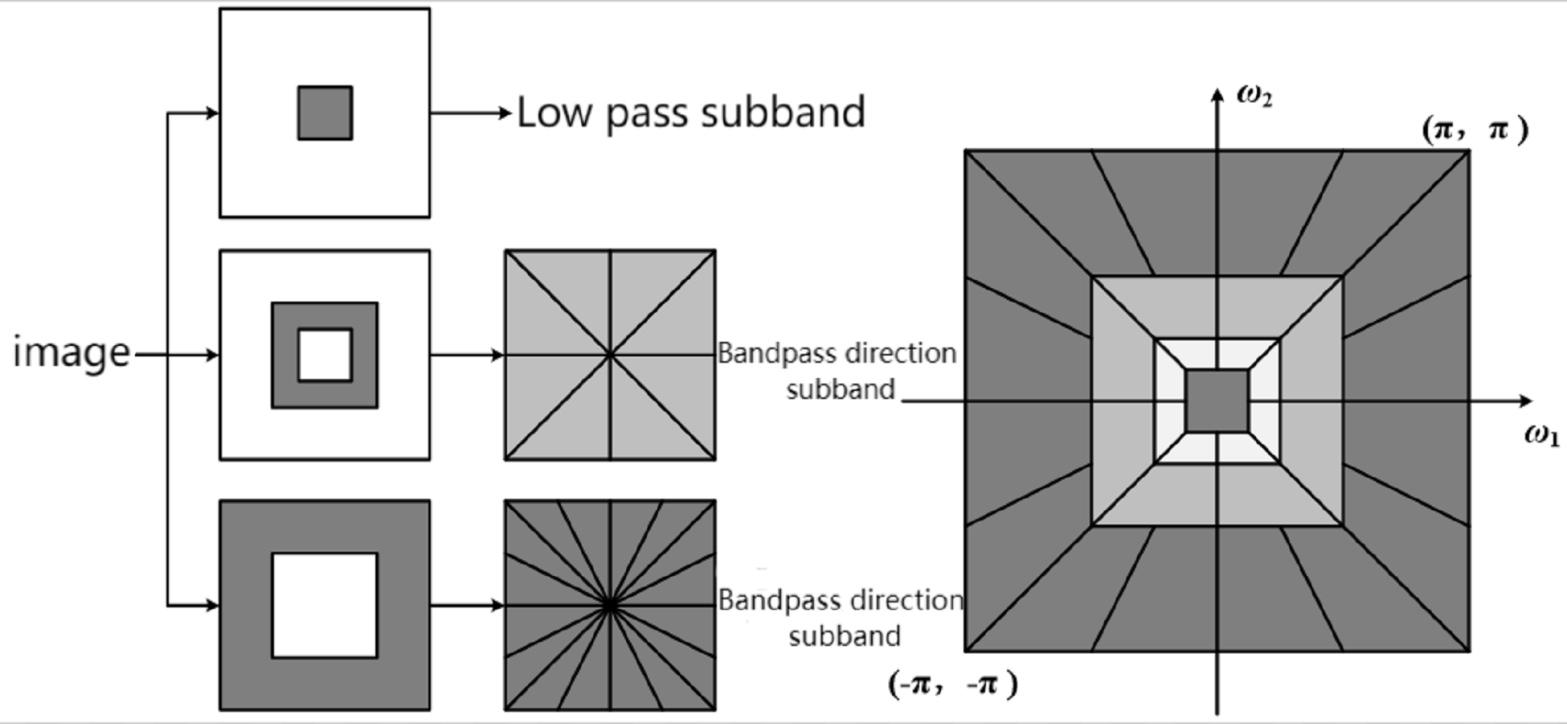
Schematic diagram of NSCT decomposition process.

Scholars have successively proposed methods to description texture feature based on time-frequency analysis. The most famous is the Laws texture (Laws, 1980). The basic idea is to first use multiple small convolution templates to filter the image to obtain features such as horizontal edges, high-frequency points, and vertical edges, and then use a larger moving window to convolve to obtain local texture energy. In terms of time-frequency analysis, after wavelet, wavelet tree and Gabor wavelet, nonsampled contourlet transform (NSCT) has gradually become an area of research interest. As shown in Fig. 2, the process is mainly divided into two steps: First, the image is divided into low-frequency subbands and multiscale high-frequency subbands with a nonsampled pyramid structure, and the multiscale decomposition results are obtained. Then, a nonsampled directional filter bank is used to decompose each high frequency subband in multiple directions. Since the filters used in the two steps are all nonsampled, the translation invariance of the filtering result is ensured.

We use nonsampled contourlet transformation to decompose the image to obtain multiscale and multidirectional subband *f*_s, d_, where *s* represents the scale and *d* represents the direction. Then, we use the local texture energy function to calculate the local energy of each scale and direction subband, and use the result as the texture feature of the image. Taking the window size as (2*n* + 1) × (2*n* + 1), the calculation formula for the texture feature *E*_s, d_ at the coordinate (*x*, *y*) in the image is as follows:

(9) \begin{eqnarray*}{E}_{s,d}(x,y)= \frac{1}{(2n+1)^{2}} \sum _{i=x-n}^{x+n}\sum _{j=y-n}^{y+n}{|}{|}{f}_{s,d}(i,j)\times g(i,j){|}-{e}_{s,d}(x,y){{|}}^{2}\end{eqnarray*}

(10) \begin{eqnarray*}{e}_{s,d}(x,y)= \frac{1}{(2n+1)^{2}} \sum _{i=x-n}^{x+n}\sum _{j=y-n}^{y+n}{|}{f}_{s,d}(i,j)\times g(i,j){|}\end{eqnarray*}

(11) \begin{eqnarray*}g(i,j)= \frac{1}{\sigma \sqrt{2\pi }} {e}^{- \frac{(i-x)^{2}+(j-y)^{2}}{2{\sigma }^{2}} }.\end{eqnarray*}

In the above formula, we believe that in a partial window, the contribution of NSCT coefficients to the texture feature of the center of the window is inconsistent. The farther from the center of the window, the smaller the contribution of the NSCT coefficient to the texture feature, and it conforms to the Gaussian distribution. It is worth mentioning that since the filter of NSCT is nonsampled, a texture feature matrix equal in size to the original image can be obtained by the method; that is, each pixel in the image has a corresponding texture feature. This provides the basis for the construction of texture heterogeneity in the next subsection.

#### Heterogeneity of texture feature

The image object has heterogeneity in spectral feature. When there are two different texture regions in an image object, the object also has heterogeneity in texture feature. Based on the remote sensing image texture feature, we propose an object-based remote sensing image MSS algorithm that combines texture features. The algorithm puts forward the concept of texture heterogeneity, and comprehensively considers the spectral feature, shape feature, and texture feature of remote sensing images, and improves the heterogeneity criterion of the MSS. Texture heterogeneity describes the degree of heterogeneity in the texture feature within an image object. Texture heterogeneity *h*_texture_ is defined as:

(12) \begin{eqnarray*}{h}_{\text{texture}}=\sum _{s,d}{\omega }_{s,d}{\sigma }_{s,d}.\end{eqnarray*}

*s* and *d* represent the scale and direction of the image texture feature, respectively. *ω*_s, d_ represents the weighting factor of the texture feature in the *s*-th scale and *d* direction, which needs to meet 0 ≤ *ω*_*s*,*d*_ ≤ 1 and ∑_*s*,*d*_*ω*_*s*,*d*_ = 1.*σ*_*s*,*d*_ represents the standard deviation of the texture feature of the image object in the *s*-th scale and *d* direction. Δ*h*_texture_ is the texture heterogeneity growth value, which is defined as follows:

(13) \begin{eqnarray*}\Delta {h}_{\text{texture}}=\sum _{s,d}{\omega }_{s,d}({n}_{\text{Merge}}\cdot {\sigma }_{s,d}^{\text{Merge}}-({n}_{\text{Obj1}}\cdot {\sigma }_{s,d}^{Obj1}+{n}_{\text{Obj2}}\cdot {\sigma }_{s,d}^{Obj2})).\end{eqnarray*}

*n*_*Obj*1_, *n*_*Obj*2_, and *n*_*Merge*_ are the area of the object *Obj1*, *Obj2* and the *merged* object respectively. ${\sigma }_{s,d}^{Obj1}$, ${\sigma }_{s,d}^{Obj2}$, and ${\sigma }_{s,d}^{Merge}$ are the standard deviation of texture feature in *s*-scale and *d*-direction of the *Obj1*,*Obj2* and the merged object respectively. Therefore, we compute the increase in the overall heterogeneity of the object *f* through the weighted sum of Δ*h*_*color*_, Δ*h*_*shape*_, and Δ*h*_*texture*_.

(14) \begin{eqnarray*}f={\omega }_{\text{color}}\cdot \Delta {h}_{\text{color}}+{\omega }_{\text{shape}}\cdot \Delta {h}_{\text{shape}}+{\omega }_{\text{texture}}\cdot \Delta {h}_{\text{texture}}.\end{eqnarray*}

*ω*_*color*_, *ω*_*shape*_, and *ω*_*texture*_ represent spectral weighting factor, shape weighting factor, and texture weighting factor, respectively, which should be meet 0 ≤ *ω*_*color*_ ≤ 1, 0 ≤ *ω*_*shape*_ ≤ 1, and *ω*_color_ + *ω*_shape_ + *ω*_texture_ = 1. Using the improved heterogeneity criterion as the merging criterion for the object-based MSS process of remote sensing images can make full use of the texture information of the features in remote sensing images and improve the segmentation accuracy of remote sensing images with rich texture information.

### Multiscale segmentation incorporating edge feature

#### Edge intensity descriptor

In the object-based MSS of remote sensing images, the shape heterogeneity criterion plays a role in controlling the shape of image objects. The shape heterogeneity criterion uses two indicators of compact heterogeneity and smooth heterogeneity to control the segmentation results. According to Eqs. (2) and (3), the algorithm considers that image objects that are closer to a circle have less compact heterogeneity, and image objects that are closer to a square have less smooth heterogeneity.

Some slender features in the image, such as rivers, roads, ditches, *etc.*, will have relatively high shape heterogeneity. When segmenting images with such features, the algorithm often divides these elongated features into segments and cannot segment similar features into complete objects. To describe the true shape of the features more effectively, we propose an edge merging cost criterion based on the edge feature of the image. Combined with the heterogeneity criterion, an object-based multiscale segmentation algorithm combining spectral, shape, texture, and edge features is proposed.

In an image, there are often obvious boundaries between areas of different types, and there are discontinuous grayscale changes at the boundaries, which constitute the edges in the image. In object-based image segmentation, once the edge of the image object is determined, the shape of the object is also determined. Common gradient operators include Roberts cross-gradient operator (Roberts & Lawrence, 1965), Prewitt operator, Sobel operator (Sobel, 1970), *etc*. Some scholars also use the feature of zero-crossing at the edge points of the second-order differential of the image and extract the edge of the image by calculating the second-order partial derivatives of the image in the *x* and *y* directions, such as the Laplacian operator. The above operators are more sensitive to noise, so it is easy to detect wrong edge points when processing noisy images.

Therefore, some scholars consider smoothing the image before detecting with the operator. Canny derived the optimal edge detection operator in two-dimensional space based on three basic criteria: low error rate, precise position and single edge point response (Canny, 1986). By considering the pros and cons of various edge detection operators, we select the Canny operator to construct the edge intensity of the image. The Canny algorithm mathematically expresses the above three criteria and tries to find the optimal solution of the expression. The edge intensity M and the normal direction *θ* of the image at the point (x, y) calculated by the Canny algorithm are:

(15) \begin{eqnarray*}M(x,y)=\sqrt{{ \left( \frac{\partial {f}_{s}}{\partial x} \right) }^{2}+{ \left( \frac{\partial {f}_{s}}{\partial y} \right) }^{2}}\end{eqnarray*}

(16) \begin{eqnarray*}\theta (x,y)=\arctan \nolimits \left( \frac{\partial {f}_{s}/\partial y}{\partial {f}_{s}/\partial x} \right) .\end{eqnarray*}

**Figure 3:**
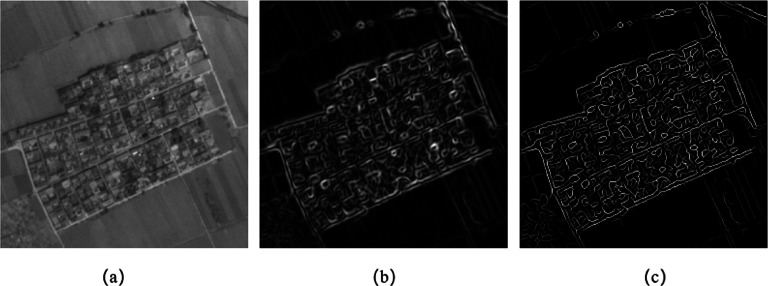
Edge intensity map. (A) Image with edge detection by canny operator. (B) Canny edge intensity map before thinning. (C) Canny edge intensity map after thinning.

Since the edges detected by the gradient operator are generally thick, it is difficult to accurately locate the edge points. The Canny algorithm uses non-maximum suppression on the edge intensity map, and detects and connects the edge points through the dual-threshold edge tracking method. The difference is that we propose to use the method of non-maximum suppression to refine the edge intensity map and use the refined result as the edge intensity map of the image. Different from the final detection result of the Canny operator, the refined edge intensity map is not a binary image, so the intensity information of the edge points is retained, which is more conducive to the use of image edge feature in subsequent processing. Figure 3A is used for edge detection using Canny operator. Figures 3B and 3C show the Canny edge intensity map before and after thinning, respectively. It can be seen in the figure that the edge in the refined edge intensity map is only one pixel wide, and its edge point positioning is more accurate.

#### Edge merging cost criterion

The algorithm puts forward the concept of “edge merging cost,” and comprehensively considers the spectral, shape, texture, and edge features of remote sensing images. In the hierarchical stepwise optimization model proposed by Beaulieu & Goldberg (1989), the image segmentation should be optimized with the smallest increase in the global expression error. The adjacent area with the smallest merging cost should be searched for each merging, and finally a multiscale expression model of the image is built. The MSS is essentially in line with the hierarchical iterative optimization model. The increase in object heterogeneity in the algorithm is the merging cost of the two objects, *i.e.,* the expression error increase expressed by the hierarchical iterative optimization model. The article defines the edge merge cost when the object Obj1 and the object Obj2 are merged as follows:

(17) \begin{eqnarray*}EdgeCost(Obj1,Obj2)=\sum _{(x,y)\in Common}M(x,y).\end{eqnarray*}

*M(x, y)* represents the edge intensity of the image in the point *(x, y)*. Common denotes a set of points adjacent to each other in the *Obj1* and *Obj2*, called adjacency edge. Common can be described as follows when four adjacency criteria are used:

(18) \begin{eqnarray*}\text{Common}=\{ (x,y)\mid (x,y)\in Obj1,\exists (x\pm 1,y\pm 1)\in {Obj}\nolimits 2\} \nonumber\\\displaystyle \bigcup \{ (x,y)\mid (x,y)\in {Obj}\nolimits 2,\exists (x\pm 1,y\pm 1)\in {Obj}\nolimits 1\} .\end{eqnarray*}

### Algorithm flow

**Figure 4:**
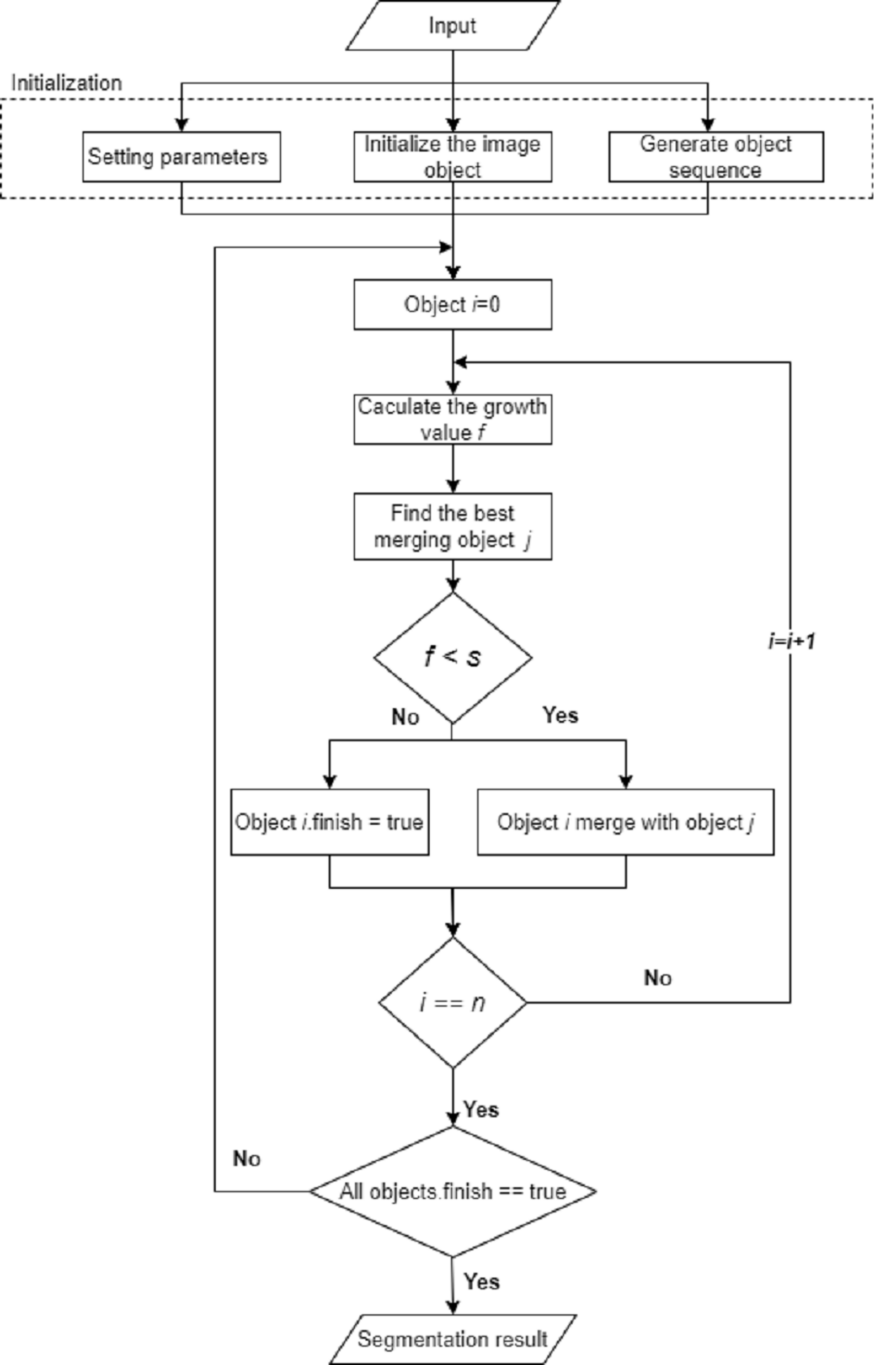
Flow chart of MSS with texture and edge features.

We propose an object-based remote sensing image MSS algorithm based on the edge merging cost criterion. The implementation process of the algorithm is shown in Fig. 4.

When computing the image object *Obj1* and the image object *Obj2* to *Obj*_Merge_, the article’s algorithm defines the merging cost *f* generated during the merging as the weighted sum of the increase value of the object heterogeneity and the edge merging cost of the object:

(19) \begin{eqnarray*}f={\omega }_{\text{color}}\cdot \Delta {h}_{\text{color}}+{\omega }_{\text{shape}}\cdot \Delta {h}_{\text{shape}}+{\omega }_{\text{texture}}\cdot \Delta {h}_{\text{texture}}\nonumber\\\displaystyle +{\omega }_{\text{edge}}\cdot {EdgeCost}\nolimits (Obj1,Obj2).\end{eqnarray*}

When performing bottom-up regional merging, the algorithm of the thesis adopts the criterion of minimum object merging cost as the regional merging strategy, combines the edge merging cost and the heterogeneity criterion to form a new merging criterion, and proposes a cost criterion based on the edge merging.

## Results

### Evaluation analysis

**Figure 5:**
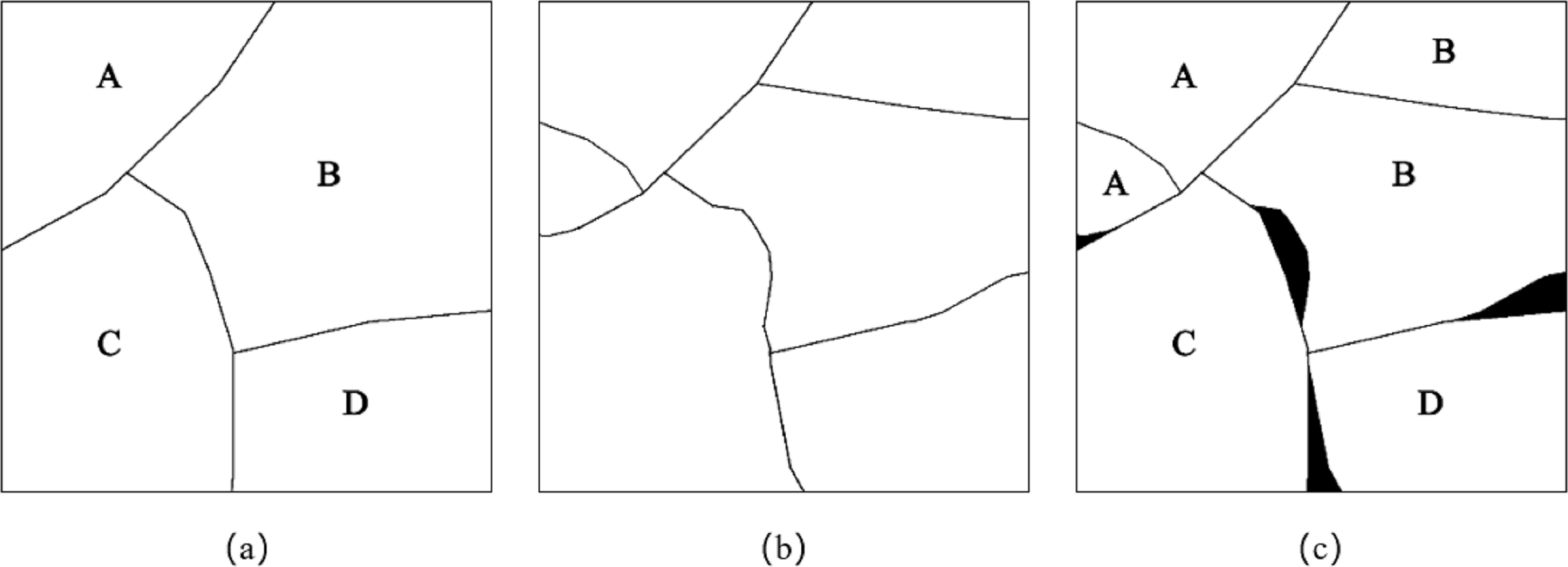
Schematic diagram of wrong segmentation based on pixel number error.

Carleer, Debeir & Wolff (2005) evaluated the segmentation accuracy through two indicators: mis-segment ratio (MR) and regions ratio (RR). Figure 5 is a schematic diagram of wrong segmentation, where Fig. 5A is the reference segmentation result, Fig. 5B is the algorithmic segmentation result, and Fig. 5C is the algorithmic wrong segmentation map, in which the black area is the wrong segmentation part.

The lower the MR, the higher the overall accuracy of image segmentation, and vice versa. The calculation formula of the wrong segmentation rate is as follows:

(20) \begin{eqnarray*}MR= \frac{\sum _{i=1}^{{N}_{\text{ref}}}\sum _{j=1}^{{N}_{\text{ref}}}{C}_{ij}-\sum _{k=1}^{{N}_{\text{ref}}}{C}_{kk}}{\sum _{i=1}^{{N}_{\text{ref}}}\sum _{j=1}^{{N}_{\text{ref}}}{C}_{ij}} \times 100\text{%}.\end{eqnarray*}

*C*_*ij*_ represents the total number of pixels whose reference category is *j* divided into category *i*, and *N*_*ref*_ represents the number of reference result regions (*i.e.,* the number of reference categories). The RR is used to comprehensively evaluate whether there is over-segmentation or under-segmentation in image segmentation. The RR is the ratio of the number *N*_*s*_ of the segmentation result area to the number *N*_*ref*_ of the reference result area. The closer the RR is to 1, the better the segmentation result. The calculation formula of the regions ratio is as follows:

(21) \begin{eqnarray*}RR= \frac{{N}_{s}}{{N}_{ref}} .\end{eqnarray*}

### Experiment 1

**Figure 6:**
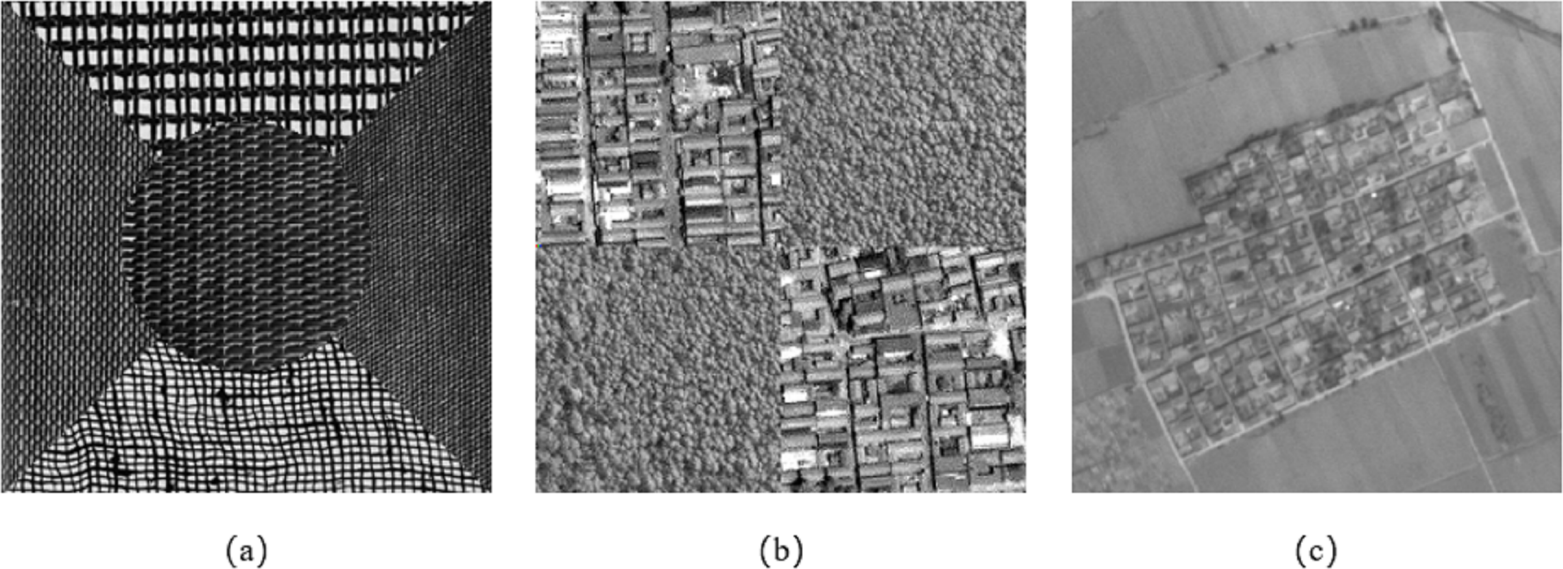
The image data selected in Experiment 1. (A) Experiment 1a: different texture composite images from the Brodatz standard texture library. (B) Experiment 2b: different texture composite images from the IKONOS satellite remote sensing image. (C) Experiment 3c: the remote sensing image of the ZY-3 satellite in Wuhan area with a spatial resolution of 2.1 m.

To verify the performance of the proposed method, we used three sets of data to test the algorithm. In the first experiment, five different textures in the Brodatz standard texture library were selected and synthesized into a texture image, as shown in Fig. 6A. In the second experiment, two typical textures in remote sensing images, architectural area and woodland, were selected and synthesized into a texture image, as shown in Fig. 6B. The remote sensing texture area is selected from the IKONOS satellite remote sensing image, and the image spatial resolution is 1 meter. The third experiment selected a part of the remote sensing image of the ZY-3 satellite in the Wuhan area with a spatial resolution of 2.1 m, as shown in Fig. 6C.

To analyze the performance of the proposed method and the MSS in segmenting images with rich texture information, we increased the weight of texture feature and reduced edge intensity. The parameters are manually tuned according to the degree of image texture and edge features. We chose the multiresolution segmentation algorithm in eCognition Developer 8.8 software (herein eCognition software) as the contrast experiments, and compared and analyzed the results of the three groups of experiments.

**Figure 7:**
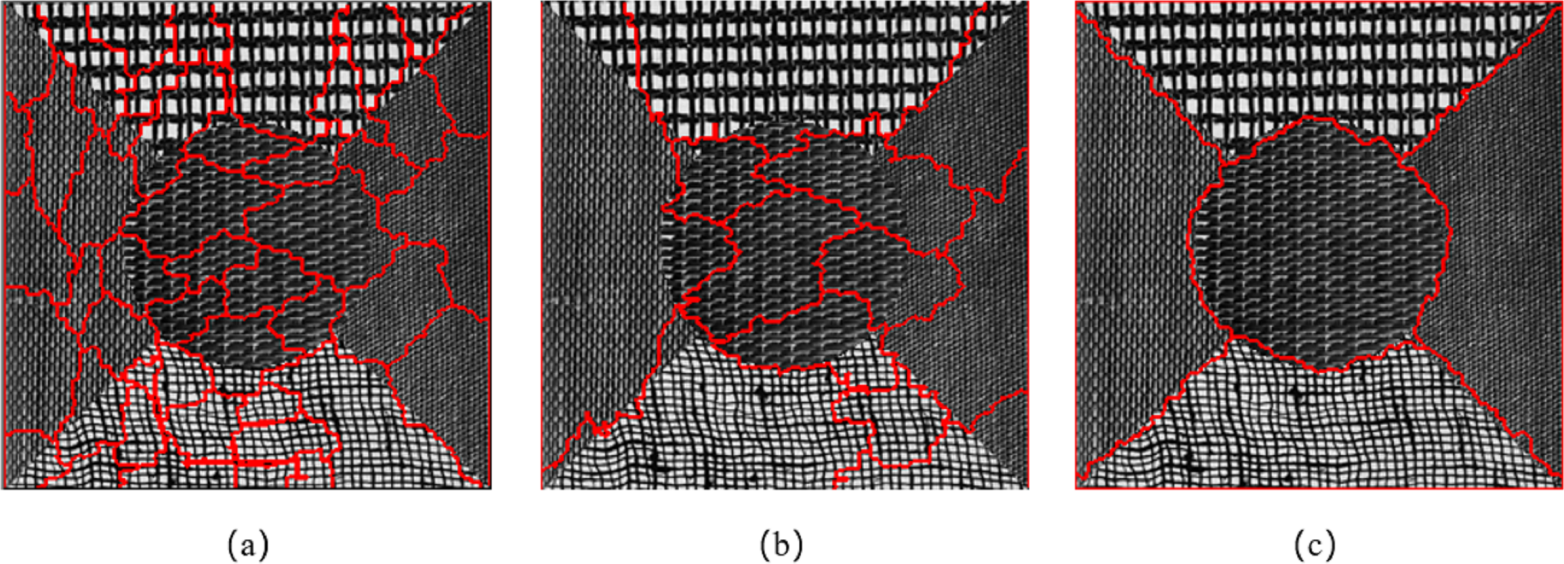
Comparison of segmentation results in Experiment 1a. (A) The segmentation result of eCognition software at fine scale. (B) The segmentation result of eCognition software at coarse scale. (C) The segmentation result of the algorithm proposed in this article.

Figure 7 shows the segmentation result of the Brodatz texture synthetic image. Figures 7A and 7B show the segmentation results of eCognition software at different scales. The parameters in Fig. 7A are set to scale *s* = 30 and weight factor *ω*_*shape*_ = 0.9 *ω*_*cmpct*_ = 0.5. The parameters in Fig. 7B are set to scale *s* = 70 and weight factor *ω*_*shape*_ = 0.8 *ω*_*cmpct*_ = 0.5. Figure 7C is the segmentation result of the algorithm proposed in this article. The parameters are set to scale *s* = 100, weight factor *ω*_*shape*_ = 0.05, *ω*_*texture*_ = 0.95, *ω*_*cmpct*_ = 0.5. It can be seen from Fig. 7 that the algorithm proposed in this article divided five different texture types into five independent and complete regions. However, the segmentation results of the eCognition software failed to obtain a complete texture area at different scales, and there is a serious mis-segmentation at the boundary of the texture area.

**Figure 8:**
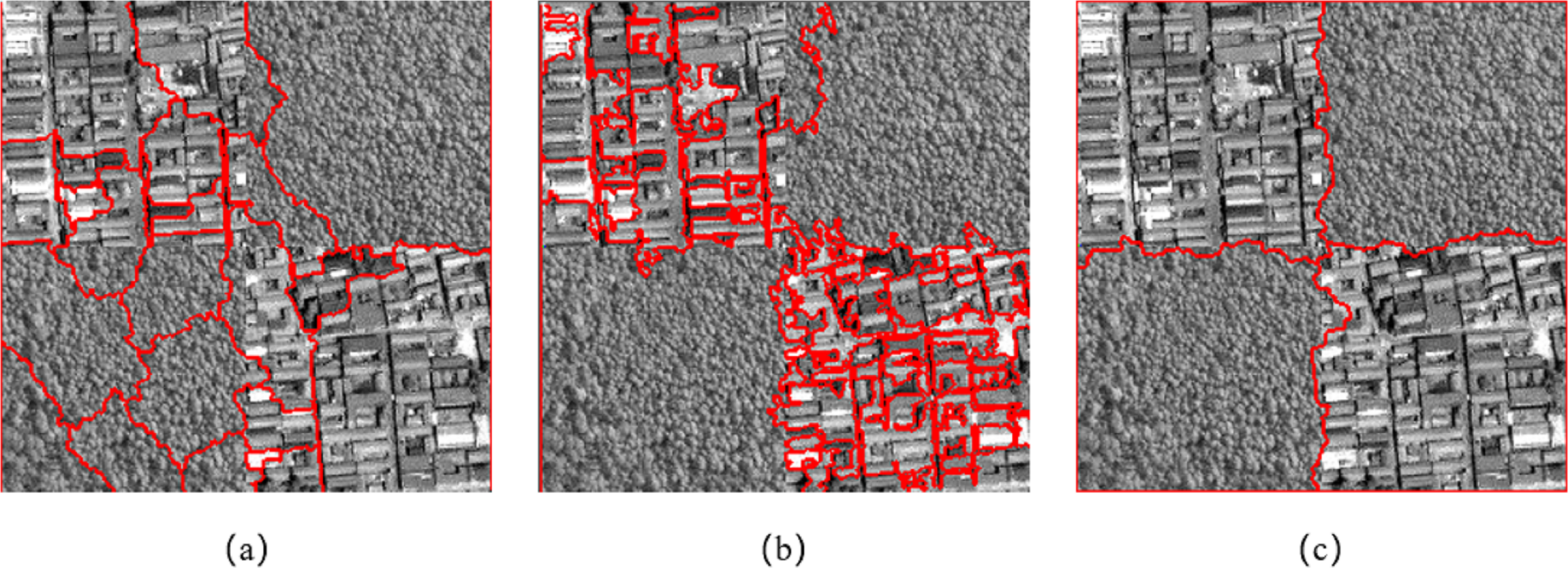
Comparison of segmentation results in Experiment 1b. (A) The segmentation result of eCognition software at fine scale. (B) The segmentation result of eCognition software at coarse scale. (C) The segmentation result of the algorithm proposed in this article.

Figure 8 shows the segmentation result of remote sensing texture synthetic image. Figures 8A and 8B are the segmentation results of eCognition software at different scales. The parameters in Fig. 8A are set to scale *s* = 50, weight factor *ω*_*shape*_ = 0.8, *ω*_*cmpct*_ = 0.5. The parameters in Fig. 8B are set to scale *s* = 80, weight factor *ω*_*shape*_ = 0.2, *ω*_*cmpct*_ = 0.5. Figure 8C is the segmentation result of the algorithm proposed in this article. The parameters are set to scale *s* = 50, weight factor *ω*_*shape*_ = 0.05, *ω*_*texture*_ = 0.95, *ω*_*cmpct*_ = 0.7. It can be seen from Fig. 8 that the algorithm proposed in this article can better distinguish the two different remote sensing textures of the construction area and the woodland and divided the image into four independent and complete areas. However, the segmentation results of the eCognition software failed to obtain a complete texture area at different scales, and there is obvious mis-segmentation at the boundary of the texture area.

**Figure 9:**
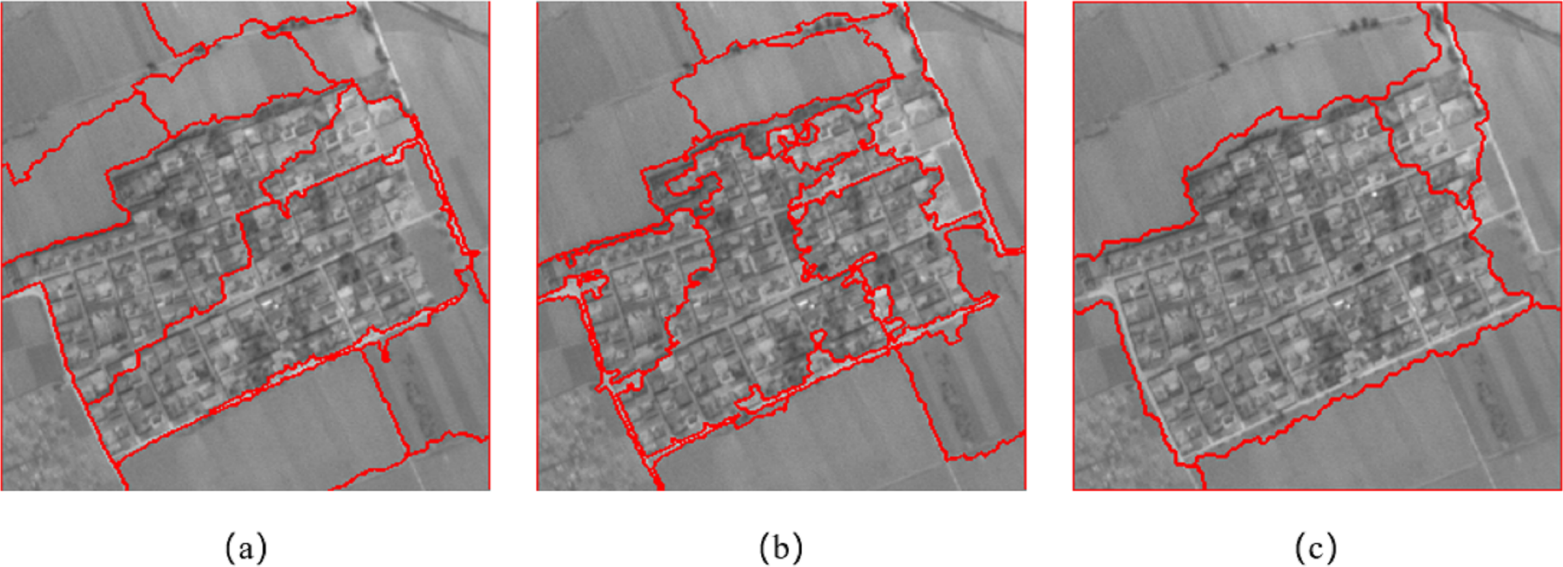
Comparison of segmentation results in Experiment 1c. (A) The segmentation result of eCognition software at fine scale. (B) The segmentation result of eCognition software at coarse scale. (C) The segmentation result of the algorithm proposed in this article.

Figure 9 shows the segmentation result of the resource No. 3 image in the Wuhan area. Figures 9A and 9B show the segmentation results of eCognition software at different scales. The parameters of Fig. 9A are set to scale *s* = 61, weight factor *ω*_*shape*_ = 0.7, *ω*_*cmpct*_ = 0.0. The parameters of Fig. 9B are set to scale *s* = 80, weight factor *ω*_*shape*_ = 0.3, *ω*_*cmpct*_ = 0.5. Figure 9C is the segmentation result of the algorithm proposed in this article. The parameters are set to scale *s* =150, weight factor *ω*_*shape*_ = 0.1, *ω*_*texture*_ = 0.8, *ω*_*cmpct*_ = 0.3. It can be seen from Fig. 9 that the algorithm proposed in this article can better distinguish the construction area in the central area of the image. The cultivated land distributed on the upper side, lower side, left side and right side of the building area is divided into four complete areas, and the building area is divided into two areas.

**Figure 10:**
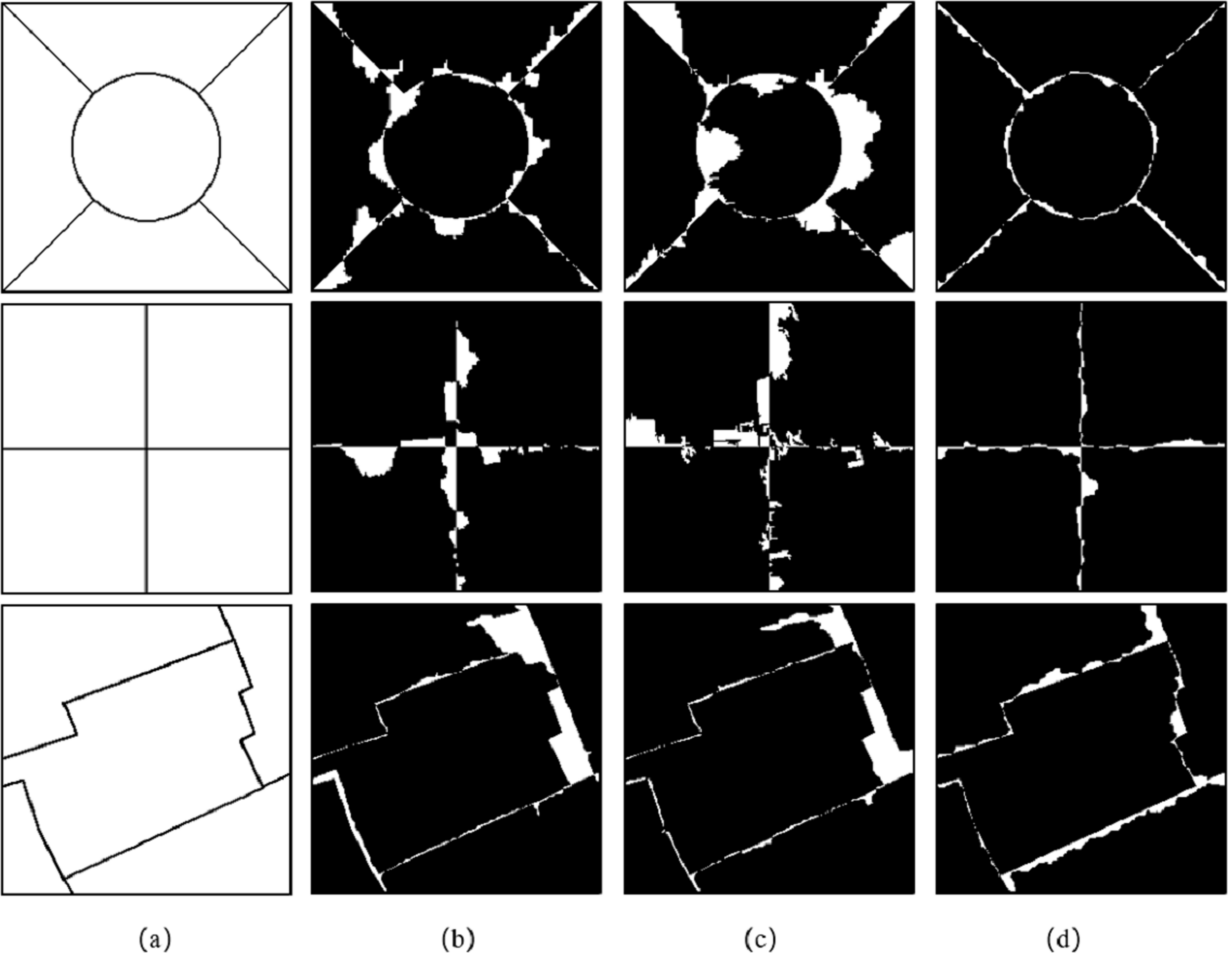
Reference segmentation results and mis-segmented diagrams of Experiment 1.

We adopted the method of supervision and evaluation, based on the error of the number of pixels, and evaluated the accuracy of the image segmentation results through two indicators: the MR and the RR. In Fig. 10, column (a) is the reference segmentation results of the three sets of experimental data, columns (b) and (c) are the mis-segmented diagrams of the eCognition software segmentation results of the three sets of experimental data. Column (d) is the mis-segmented map of the segmentation result of the algorithm proposed in this article, in which the white part is the mis-segmented pixel.

Table 1 evaluates the segmentation results of Experiment 1. It can be seen from Table 1 that the MR of the segmentation results of the proposed algorithm for the three groups of experiments in the article are lower than the segmentation results obtained using eCognition software.

**Table 1:** Accuracy evaluation of Experiment 1 segmentation results.

	**eCognition algorithm (fine scale)**			**eCognition algorithm (coarse scale)**			**Algorithm of article**		
	**Mis-segment ratio (%)**	**Regions ratio**	**scale**	**Mis-segment ratio (%)**	**Regions ratio**	**scale**	**Mis-segment ratio (%)**	**Regions ratio**	**scale**
Brodatz texture composite image	8.36	10.2	30	14.35	2.4	70	3.23	1	100
Remote sensing texture synthesis image	5.65	6	50	6.47	8.75	80	2.48	1	50
Resource No. 3 image	7.41	2.8	60	5.84	3	80	4.68	1.2	150

Therefore, in the three sets of experiments, the eCognition software has different degrees of mis-segmentation at the boundary of the image texture area. In the later experiments, for the more complex texture area of the building area, the eCognition software has the phenomena of over-segmentation at different scales.

In summary, because the regions with rich texture information in the image show different spectral heterogeneity in the spectral feature, the traditional object-based MSS algorithm does not fully consider the texture information of the features in the image. Therefore, over-segmentation will occur when segmenting images with rich texture information.

### Experiment 2

**Figure 11:**
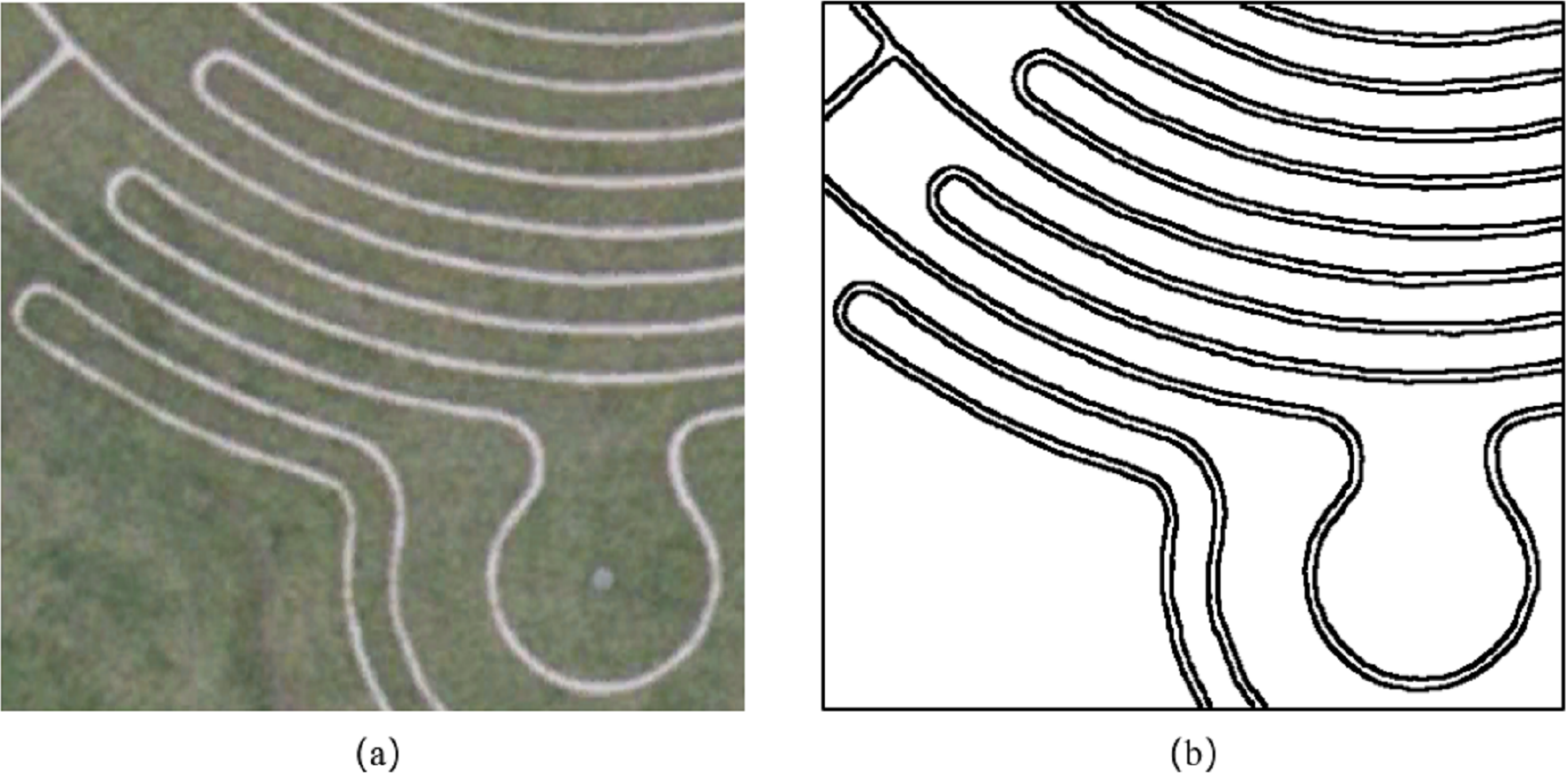
Experiment 2 image and reference segmentation results.

When segmenting images with less texture information and rich edge information, we increased the weight of the edge intensity and reduced the weight of the texture information. We selected a satellite remote sensing image of Milton Keynes, UK, as shown in Fig. 11A; the image was acquired in 2005. The area covered in the image includes the corner of a plant maze, and there are two dominant types of features, grass and roads. Through visual interpretation, we manually segmented the image and extracted eight roads and 10 grasslands separated by roads, totaling 18 areas. The reference segmentation result is shown in Fig. 11B. At the same time, we choose the MSS algorithm in eCognition software as a comparative experiment.

**Figure 12:**
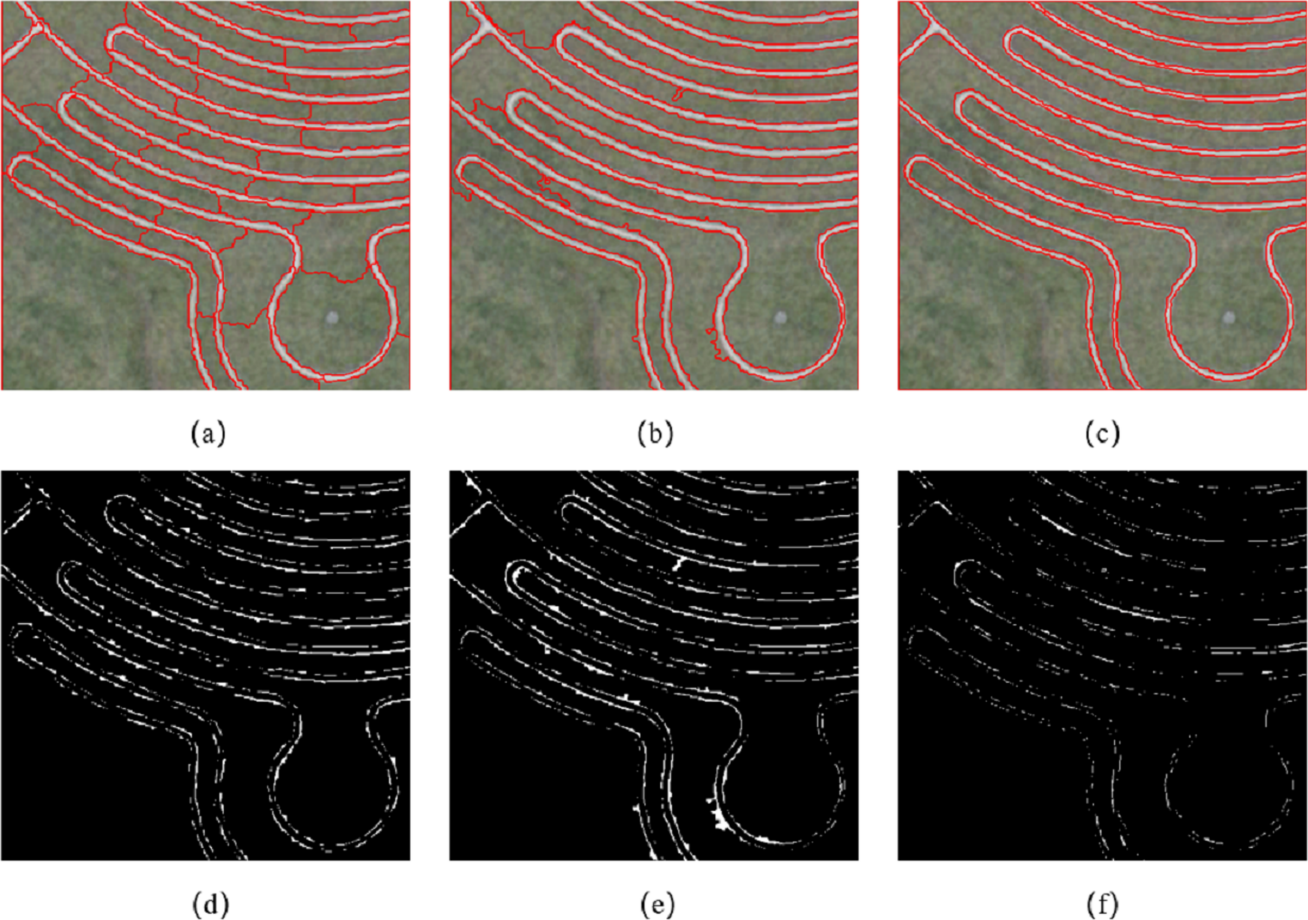
Experiment 2 image segmentation result comparison.

Figure 12 is the segmentation result of the first set of experiments. Figures 12A and 12B are the segmentation results by using the eCognition. The parameters of Fig. 12A are *s* = 70, *ω*_*shape*_ = 0.8, *ω*_*cmpct*_ = 0.5, while the parameters of Fig. 12B are *s* =150, *ω*_*shape*_ = 0.1, *ω*_*cmpct*_ = 0.5. Figure 12C is the segmentation result of this article’s algorithm, and the parameters are *s* =150, *ω*_*color*_ = 0.5, *ω*_*edge*_ = 0.5. Figures 12D, 12E and 12F indicates incorrect segmentation results of eCognition and our algorithm.

It can be seen from Fig. 12 that the algorithm proposed in the article divides the eight roads and 10 grasslands in the image into independent and complete objects. For the segmentation results of the eCognition software, when the shape weighting factor is large, the software segmented the road and grass in the image into several segments, failing to segment the complete objects, as shown in Fig. 12A. When the shape weighting factor is small, because the algorithm’s constraints on the shape feature are weakened, the road objects in the segmentation result have some burrs at different positions, as shown in Fig. 12B. The proposed algorithm made full use of the edge feature in the remote sensing image, and the location of the edge feature is more accurate.

Based on the error of the number of pixels, we evaluated the accuracy of the image segmentation results through two indicators: the MR and the RR. Table 2 shows the segmentation accuracy of Experiment 2 image by different algorithms. It can be seen from the table that the ratio of the segmentation results of eCognition software at different scales is greater than 1, and there are different degrees of over-segmentation. When the scale *s* = 150, the RR of the eCognition software segmentation result is closer to 1, but due to the lack of shape constraints, the positioning of the edge feature is inaccurate, and the MR is slightly higher than the result of the scale *s* = 70. The proposed algorithm divides the regions with similar features into independent and complete objects, the RR is equal to 1, and the MR is lower than the segmentation results of eCognition software at different scales.

**Table 2:** Accuracy evaluation of Experiment 2 segmentation results.

	**eCognition algorithm (fine scale)**	**eCognition algorithm (coarse scale)**	**Proposed method**
Mis-segment ratio (%)	4.22	4.42	1.44
Regions ratio	3.06	1.22	1

### Experiment 3

When segmenting images with rich texture information and edge information, we adaptively adjust the weights of textures and edges.

**Figure 13:**
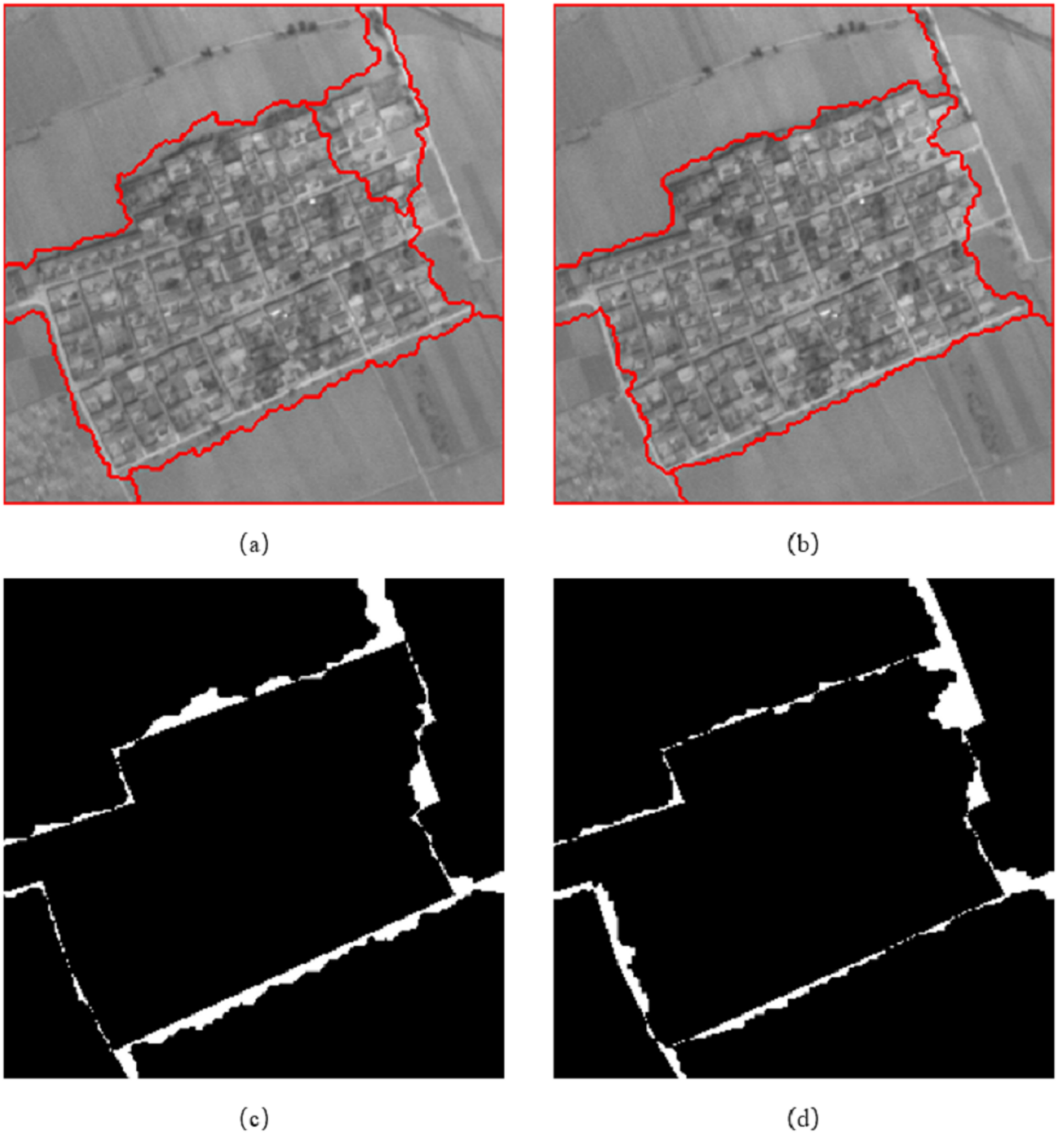
Comparison of the results of image segmentation of resource No. 3.

Figure 13A is the segmentation result of the algorithm proposed in Experiment 1, and Fig. 13B is the result of the object-based segmentation algorithm based on the edge merging cost criterion proposed in this section. The parameters in Fig. 13A are set to scale *s* =150 and weight factor *ω*_*shape*_ = 0.1, *ω*_*cmpct*_ = 0.3, *ω*_*texture*_ = 0.8. The parameters in Fig. 13B are set to scale *s* =150 and weight factor *ω*_*shape*_ = 0.1, *ω*_*cmpct*_ = 0.3, *ω*_*texture*_ = 0.7, *ω*_*edge*_ = 0.2. Figures 13C and 13D are the corresponding mis-segmentation diagrams, respectively.

It can be seen from Fig. 13 that after the algorithm incorporates the edge feature, the accuracy of the boundary location of the texture area has been significantly improved, and the upper and lower boundaries of the building area can be found more accurately, and the building is divided into an independent and complete area. Table 3 shows the segmentation accuracy of the two algorithms. It can be seen from the table that the segmentation result of the algorithm based on the edge merging cost criterion is lower in the error segmentation rate than the object-based segmentation algorithm combined with the texture feature, and the RR is equal to 1.

**Table 3:** Accuracy evaluation of Experiment 3 segmentation results.

	**Method combined with texture features**	**Method combined with texture features** **and edge merging cost criterion**
Mis-segment ratio (%)	4.68	4.48
Regions ratio	1.2	1

Through three sets of experiments, the article supports the effectiveness of the object-based MSS algorithm based on the edge merging cost criterion. While using the spectral, shape, and texture features of remote sensing images, the algorithm accounts for the edge feature of the image. The segmentation results can accurately locate the boundaries of the features, and its segmentation accuracy is better than traditional algorithms.

## Conclusions

This article takes the MSS as an example, focusing on the framework and process of object-based MSS algorithms. In response to texture feature and edge feature of the ground features in remote sensing images are being rarely used, the specific improvements proposed in this article are as follows:

1. We proposed a remote sensing image texture feature description method based on time-frequency analysis, which constructs texture heterogeneity. Additionally, a combined texture feature-based object-based MSS algorithm is proposed. Experiments demonstrated that the algorithm can better distinguish different textures in remote sensing images, and it has a better segmentation effect on images with rich texture information.
2. We used the Canny operator to describe the edge intensity of remote sensing images, and proposed an edge merging cost criterion. Experiments demonstrated that the algorithm can locate the boundaries of features in remote sensing images more accurately, and it has a better segmentation effect on remote sensing images with rich texture information.

Through the algorithm proposed in this article, when segmenting features with rich texture information and slender shaped features, a more complete segmentation object can be obtained, and over segmentation does not occur easily, which will be more conducive to follow up processing and analysis of remote sensing images. The proposed object-based MSS algorithm proposed can effectively obtain more complete ground objects, thereby solving the problem of determining objects in the visual attention model. The research results can be widely used in objected building extraction from high-resolution remote sensing images. A limitation of this study is that we do not use the more popular deep learning method, but use a more traditional segmentation algorithm, so how to combine the deep learning method is a direction for future research.

## Supplemental Information

10.7717/peerj-cs.1290/supp-1 Supplemental Information 1 Code of the object-based multiscale segmentation Method incorporating texture and edge features The program is written in C++ language, and use a graphical user interface to read, process and show the experimental images automatically. The parameters can be set from the parameter dialog box.

10.7717/peerj-cs.1290/supp-2 Supplemental Information 2 Data of the object-based multiscale segmentation Experiments incorporating texture and edge features The input data, process data and result data. “Figure6a.bmp” is the raw data for Figure6(a). “Figure6b.bmp” is the raw data for Figure6(b). “Figure6c.bmp” is the raw data for Figure6(c). “Figure11.bmp” is the raw data for Figure11.

## Additional Information and Declarations

### Data Availability

The following information was supplied regarding data availability: The raw measurements are available in the Supplementary Files 1 and 2.
