# Supplementary material for: Object-based multiscale segmentation incorporating texture and edge features of high-resolution remote sensing images
Source: PeerJ Comput Sci. 2023 Mar 15;9:e1290. doi: 10.7717/peerj-cs.1290 (PMC10280506; doi:10.7717/peerj-cs.1290)
Supplement: Supplemental Information 1 — The program is written in C++ language, and use a graphical user interface to read, process and show the experimental images automatically. The parameters can be set from the parameter dialog box. [file peerj-cs-09-1290-s001.zip › cs-79984-Image_segmentation_code/segment.plg]

```
# Build Log


### --------------------Configuration: segment - Win32 Release--------------------


### Command Lines


### Results

segment.exe - 0 error(s), 0 warning(s)
```
