# Supplementary figures and images for: Object-based multiscale segmentation incorporating texture and edge features of high-resolution remote sensing images
Source: PeerJ Comput Sci. 2023 Mar 15;9:e1290. doi: 10.7717/peerj-cs.1290 (PMC10280506; doi:10.7717/peerj-cs.1290)

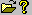

Supplement: Supplemental Information 1 — The program is written in C++ language, and use a graphical user interface to read, process and show the experimental images automatically. The parameters can be set from the parameter dialog box. [file peerj-cs-09-1290-s001.zip › cs-79984-Image_segmentation_code/res/Toolbar.bmp]

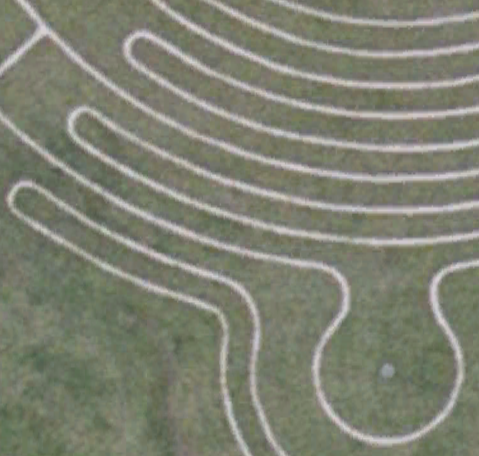

Supplement: Supplemental Information 2 — The input data, process data and result data. “Figure6a.bmp” is the raw data for Figure6(a). “Figure6b.bmp” is the raw data for Figure6(b). “Figure6c.bmp” is the raw data for Figure6(c). “Figure11.bmp” is the raw data for Figure11. [file peerj-cs-09-1290-s002.zip › cs-79984-Image_segmentation_data/Figure11.bmp]

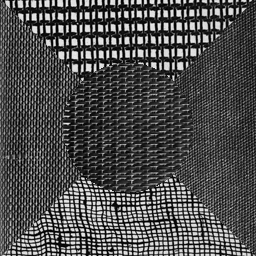

Supplement: Supplemental Information 2 — The input data, process data and result data. “Figure6a.bmp” is the raw data for Figure6(a). “Figure6b.bmp” is the raw data for Figure6(b). “Figure6c.bmp” is the raw data for Figure6(c). “Figure11.bmp” is the raw data for Figure11. [file peerj-cs-09-1290-s002.zip › cs-79984-Image_segmentation_data/Figure6a.bmp]

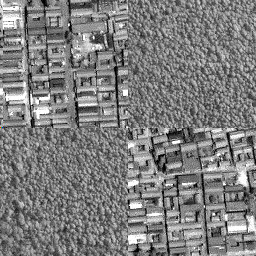

Supplement: Supplemental Information 2 — The input data, process data and result data. “Figure6a.bmp” is the raw data for Figure6(a). “Figure6b.bmp” is the raw data for Figure6(b). “Figure6c.bmp” is the raw data for Figure6(c). “Figure11.bmp” is the raw data for Figure11. [file peerj-cs-09-1290-s002.zip › cs-79984-Image_segmentation_data/Figure6b.bmp]

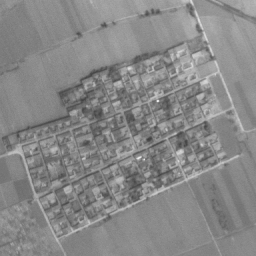

Supplement: Supplemental Information 2 — The input data, process data and result data. “Figure6a.bmp” is the raw data for Figure6(a). “Figure6b.bmp” is the raw data for Figure6(b). “Figure6c.bmp” is the raw data for Figure6(c). “Figure11.bmp” is the raw data for Figure11. [file peerj-cs-09-1290-s002.zip › cs-79984-Image_segmentation_data/Figure6c.bmp]
